# Hemistepsin A suppresses colorectal cancer growth through inhibiting pyruvate dehydrogenase kinase activity

**DOI:** 10.1038/s41598-020-79019-1

**Published:** 2020-12-14

**Authors:** Ling Jin, Eun-Yeong Kim, Tae-Wook Chung, Chang Woo Han, So Young Park, Jung Ho Han, Sung-Jin Bae, Jong Rok Lee, Young Woo Kim, Se Bok Jang, Ki-Tae Ha

**Affiliations:** 1 Department of Korean Medical Science, School of Korean Medicine, Pusan National University, Busandaehak-ro 49, Yangsan, Gyeonsangnam-do 50612 Republic of Korea; 2 Healthy Aging Korean Medical Research Center, Pusan National University, Yangsan, Gyeongsangnam-do 50612 Republic of Korea; 3 Department of Molecular Biology, College of Natural Sciences, Pusan National University, Geumjeong-gu, Busan, 46241 Republic of Korea; 4 Department of Pharmaceutical Engineering, Daegu Haany University, Gyeongsan, Gyeongsangbuk-do 38610 Republic of Korea; 5 School of Korean Medicine, Dongguk University, Gyeongju, 38066 Korea

**Keywords:** Cancer, Cell biology, Chemical biology, Drug discovery

## Abstract

Most cancer cells primarily produce their energy through a high rate of glycolysis followed by lactic acid fermentation even in the presence of abundant oxygen. Pyruvate dehydrogenase kinase (PDK) 1, an enzyme responsible for aerobic glycolysis via phosphorylating and inactivating pyruvate dehydrogenase (PDH) complex, is commonly overexpressed in tumors and recognized as a therapeutic target in colorectal cancer. Hemistepsin A (HsA) is a sesquiterpene lactone isolated from *Hemistepta lyrata* Bunge (Compositae). Here, we report that HsA is a PDK1 inhibitor can reduce the growth of colorectal cancer and consequent activation of mitochondrial ROS-dependent apoptotic pathway both in vivo and in vitro. Computational simulation and biochemical assays showed that HsA directly binds to the lipoamide-binding site of PDK1, and subsequently inhibits the interaction of PDK1 with the E2 subunit of PDH complex. As a result of PDK1 inhibition, lactate production was decreased, but oxygen consumption was increased. Mitochondrial ROS levels and mitochondrial damage were also increased. Consistent with these observations, the apoptosis of colorectal cancer cells was promoted by HsA with enhanced activation of caspase-3 and -9. These results suggested that HsA might be a potential candidate for developing a novel anti-cancer drug through suppressing cancer metabolism.

## Introduction

Colorectal cancer (CRC) is the third most commonly diagnosed malignant cancer and the fourth leading cause of cancer-associated deaths in the world^1^. Cancer cells generate their energy through a high level of glycolysis, rather than relying on mitochondrial oxidative phosphorylation (OXPHOS) even in the presence of oxygen^2,3^. Although cancer is heterogeneous and many cancers have been found to rely on both glycolysis and OXPHOS^4^, upregulated glycolysis was reported as a prognostic factor of CRC^5^. Thus, targeting aerobic glycolysis, i.e. Warburg effect, is expected to be a promising strategy against CRC.

Four different isoforms of pyruvate dehydrogenase kinase (PDK) have been reported to be variably overexpressed in different types of cancer. The PDKs inactivate the pyruvate dehydrogenase (PDH) complex by phosphorylating its E1α subunit (PDHA1)^6^. Suppression of PDH by PDKs prevents the conversion of pyruvate into acetyl-CoA; instead, the pyruvate is converted into lactate^7^. PDK1, which phosphorylates all three sites of PDHA1 (S232, S293, and S300)^8^, is frequently overexpressed in cancer including gastric cancer, acute myeloid leukemia, non-small cell lung cancer, and CRC^9–12^. Previous studies have also shown that inhibition of PDK1 activity using a small molecule inhibitor can suppress the proliferation and growth of CRC^11,12^. Therefore, to regulate the Warburg effect, we focus on targeting pyruvate dehydrogenase kinase (PDK) 1. Previously, our group identified several novel PDK1 inhibitors, including Huzhangoside A isolated from *Anemone rivularis* and Ilimaquinone from *Smenospongia cerebriformis*^13,14^.

Hemistepsin A (HsA) is a sesquiterpene lactone isolated from *Hemistepta lyrata* Bunge which has been used for the treatment of colon diseases, such as diarrhea and anal fistula. HsA has been reported for its anti-tumor property against several tumor types^15^. However, the precise mechanism of the anti-tumor effect or the in vivo efficacy of HsA on CRC had not been explored. In this study, we demonstrated that HsA has an inhibitory effect on PDK1 activity, and it subsequently induces mitochondrial reactive oxygen species (ROS)-mediated apoptosis of CRC cells in both in vitro and in vivo studies.

## Results

### HsA shows cytotoxic effects on colorectal cancer cells in vitro

**Figure 1:**
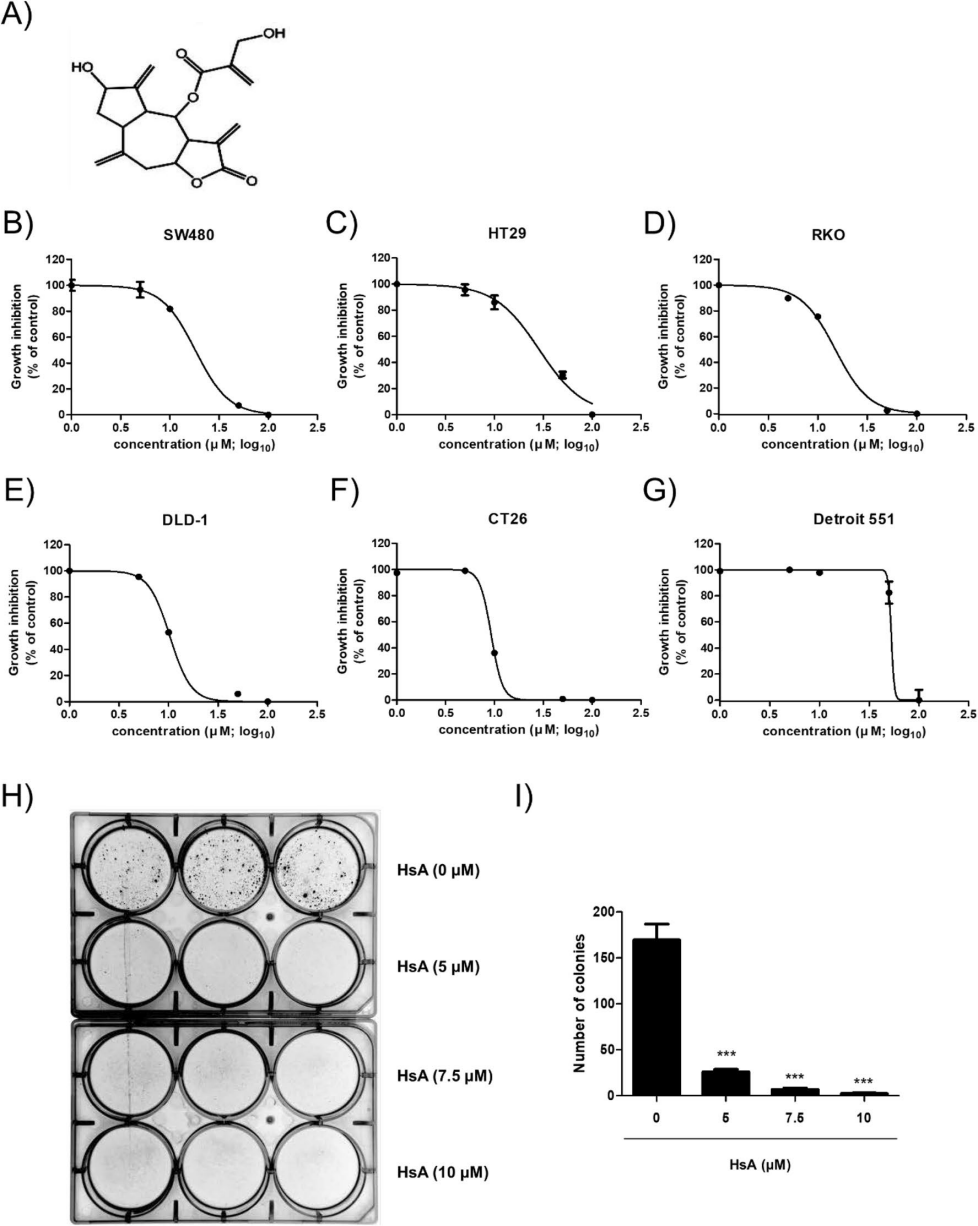
Chemical structure and cytotoxic effect of HsA. (**A**) The chemical structure of HsA (CID: 10043230). (**B**–**G**) SW480 (**B**), HT29 (**C**), RKO (**D**), DLD-1 (**E**), CT26 (**F**), and Detroit 551 (**G**) cells were treated with the indicated concentrations of HsA (0, 1, 5, 10, 50, 100 μM) for 24 h. The cytotoxic effects of HsA were measured by MTT Assay. All results were shown as log concentration calculating IC50from a dose–response curve compared with the control group. (**H**) DLD-1 cells were cultured with HsA (0, 5, 7.5, 10 μM) for 2 weeks, and colonies were analyzed. Results are presented as means ± SEM. ***p < 0.001, compared to the control.

The chemical structure of HsA illustrated in Fig. 1A was already confirmed by nuclear magnetic resonance spectroscopy^16^. The in vitro cytotoxic effect of HsA was measured in several CRC cells, including SW480, HT29, RKO, DLD-1, and murine CT26. HsA showed significant cytotoxicity in the CRC cells at a concentration of 50 μM at 24 h time point. However, under the same conditions, HsA showed much lower cytotoxicity in normal human fibroblast Detroit 551 cells (Fig. 1B–G). We also calculated the IC_50_ of HsA assuming the concentration of HsA inhibits the growth of 50% of each cell line and found that DLD-1 cells were most sensitive to HsA among the human colon cancer cell lines (Table S2). Then, we observed the long-term effect of HsA in DLD-1 cells (Fig. 1H–I). HsA significantly reduced the number of surviving colonies of DLD-1 cells. Thus, the human DLD-1 colon adenocarcinoma cells were selected for further in vitro assays.

### HsA suppresses lactate production and reduces the phosphorylation of PDHA1

**Figure 2:**
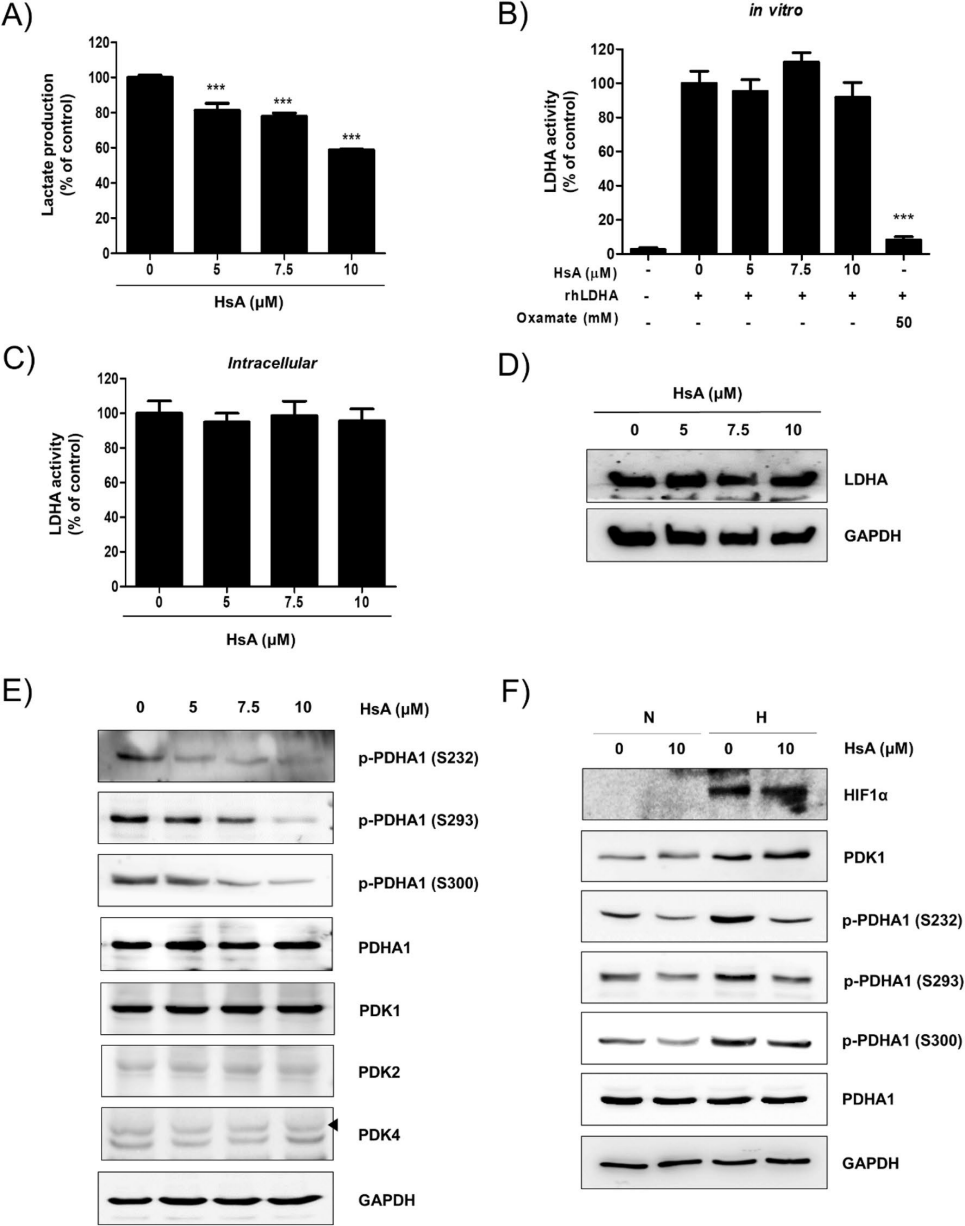
HsA suppresses the lactate production by inhibiting the PDK1 activity. (**A**) DLD-1 cells were treated with the indicated concentrations of HsA (0, 5, 7.5, 10 μM) for 4 h. Production of lactate in DLD-1 cell culture media was determined using a commercially available lactate assay kit. (**B**) In vitro LDHA activity was estimated using recombinant human LDHA with the indicated concentration of HsA (0, 5, 7.5, 10 μM) or Oxamate (50 mM). (**C**) DLD-1 cells were incubated with the indicated concentrations of HsA (0, 5, 7.5, 10 μM) for 4 h. The cells were lysed and LDHA activities were measured using the cell lysates as an enzyme source. (**D**) DLD-1 cells were cultured with the indicated concentrations of HsA (0, 5, 7.5, 10 μM) for 4 h. The levels of LDHA were examined by Western blot analysis. GAPDH expression was used as an internal loading control. (**E**) DLD-1 cells were cultured with the indicated concentrations of HsA (0, 5, 7.5, 10 μM) for 4 h. The levels of phosphorylated PDHA1 (S232, S293 and S300), total PDHA1, PDK1, PDK2, and PDK4 were examined by Western blot analysis. GAPDH expression was used as an internal loading control. The results (**A**, **B**) are shown as mean + SEM. ***p < 0.001 compared with the control group. (**F**) DLD-1 cells were treated with HsA (0, 10 μM) under normoxic or hypoxic conditions for 4 h. The levels of HIF1α, the phosphorylated PDHA1 (S232, S293, and S300), total PDHA1, and PDK1 were examined by Western blot analysis. GAPDH expression was used as an internal loading control.

The human colon cancer DLD-1 cells and murine colorectal cancer CT26 cells were treated with HsA for 4 h. HsA did not show any cytotoxic effect at a concentration of 10 μM in both DLD-1 and CT26 cells (Fig. S1). Thus, several in vitro assays were performed at the conditions. The amount of extracellular lactate was significantly decreased upon HsA treatment in a dose-dependent manner (Fig. 2A). To identify how HsA reduces lactate production, the activities and expressions of LDHA and PDKs, two major enzymes involved in converting pyruvate into lactate^17^, were examined. As shown in Fig. 2B–D HsA did not show a significant effect on in vitro and intracellular LDHA activity nor its expression. On the other hand, the HsA treatment markedly reduced the phosphorylation of PDHA1 in DLD-1 and CT 26 cells (Figs. 2E and S2). However, HsA did not affect the expression of the PDK isotypes, such as PDK1, PDK2, PDK3, and PDK4, in both cell lines. The expression of PDK3 was not detected in DLD-1 cells (data not shown). We tested whether HsA has an inhibitory effect on hypoxia-induced phosphorylation of PDHA1. As shown in Fig. 2F, hypoxia increased PDK1 and p-PDHA (S232, S293, and S300) expression. However, increased p-PDHA1 levels were decreased by HsA treatment. These results indicate that HsA reduces the phosphorylation of PDHA1 by reducing the activity of PDK1, but not affecting its expression.

### HsA decreases PDK1 activity by interfering with the lipoamide-binding domain of the PDH E2 subunit (PDH-E2)

**Figure 3:**
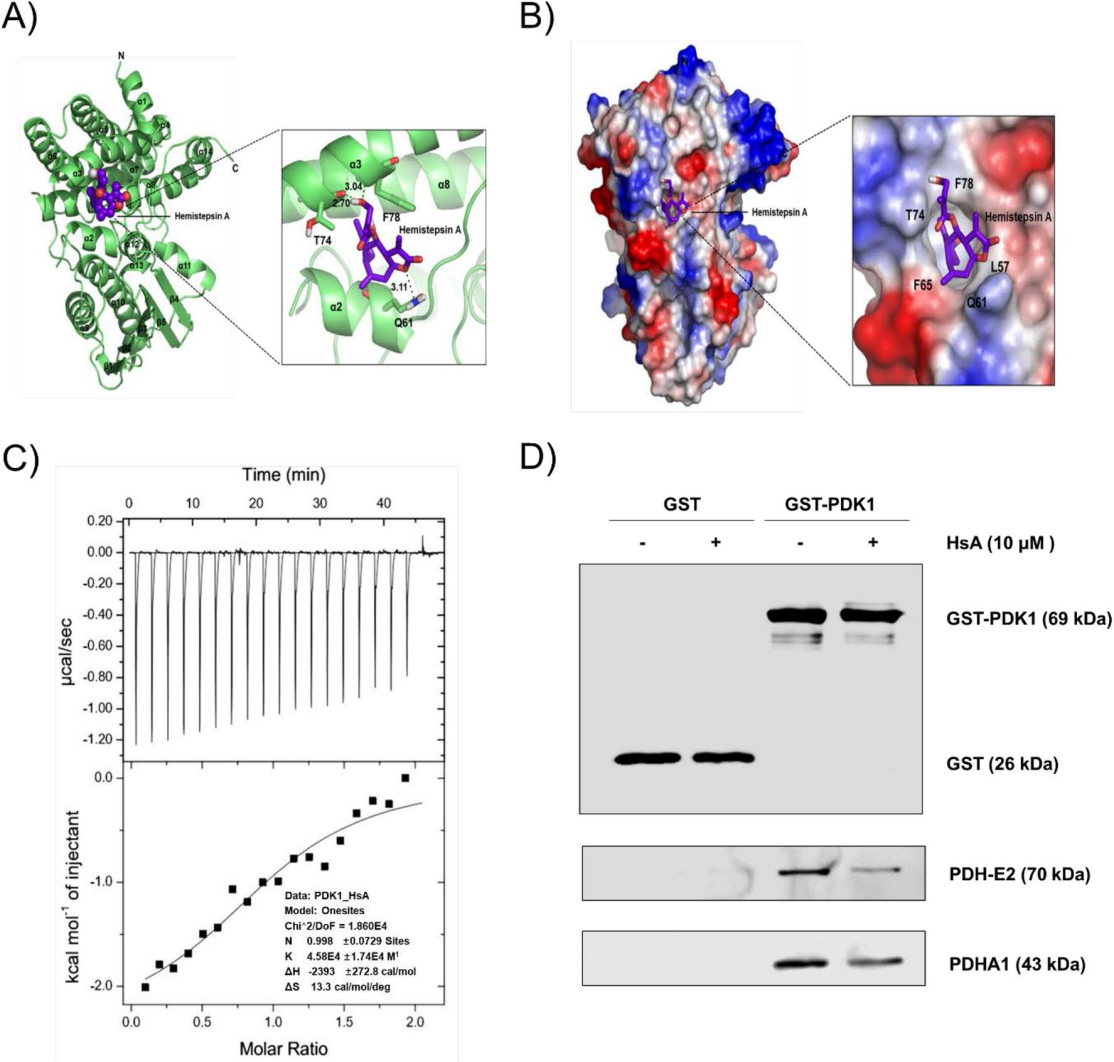
HsA decreases the PDK activity by binding to the lipoamide-binding domain of PDK1. Predicted structure of PDK1 with HsA (**A**) The modeled structure of PDK1 with HsA is shown as a ribbon representation. The interaction residues between PDK1 and HsA are shown and hydrogen bonds are shown as black dotted lines. (**B**) The complex structure of PDK1 with HsA is shown as a surface representation. The relative distribution of the electrostatic surface of PDK1 is shown with the acidic region in red, basic region in blue, and neutral region in white. (**C**) ITC analysis of the PDK1 and HsA interaction is shown. HsA was titrated with PDK1 solution. (**D**) HEK 293T cells transfected with GST and GST-PDK1 were treated with HsA (0, 10 μM) for 12 h. GST and GST-PDK1 fused proteins were pulled down by GST-tagged beads, followed by incubation with whole cell lysates of HEK 293 T cells. The beads were run on SDS-PAGE gel and the levels of PDH-E2 and PDHA1 were measured by Western blot analysis. The amounts of GST and GST-PDK1 were used as a loading control.

To elucidate the mechanism underlying the inhibitory effect of HsA on PDK1 activity, the interaction and the binding affinity between PDK1 and HsA was analyzed by in silico modeling. The modeled structure of PDK1 with HsA has shown with ribbon and surface representations indicated that HsA bound to Phe78, Gln61 and Thr74 residues of PDK1 (Fig. 3A,B; Lys246 and Asn247 residues of PDK2; Tyr155, Gly325 and Tyr326 residues of PDK3; Arg170, Glu254 and Tyr340 residues of PDK4 (Fig. S3). Among the PDK isoforms, HsA was predicted to bind adjacent to the hydrophobic residues of PDK1 (Leu57, Phe65, and Phe78), which residues participating in the lipoamide-binding pocket^18^. Next, an isothermal titration calorimeter (ITC) approach was used to confirm the binding ability of HsA and PDK1. The results showed that HsA bound physically to PDK1 with an apparent dissociation constant (K_D_) of 22 μM (Fig. 3C). To elucidate the precise mode of binding inhibition, the binding affinities between PDK1 protein and lipoamide-binding domains of PDH-E2 (L1 and L2) with or without HsA were measured by ITC. The K_D_ values for the PDK1 interactions with the ligands (L1, L1-HsA, L2, and L2-HsA) were determined to be 41.15 μM, 43.10 μM, 30.03 μM, and 40.98 μM, respectively (Fig. S4). The results indicated that the interaction between PDK1 and the L2 domain of PDH-E2 was reduced in the presence of HsA. To confirm the in vitro interaction data, an intracellular interaction assay between PDK1 and PDH-E2 was conducted using the overexpression of glutathione S-transferase (GST) tagged-PDK1. The results demonstrated that HsA decreased the association of GST tagged-PDK1 with PDH-E2 (Fig. 3D). Consequently, the interaction of PDK1 with PDHA1 proteins was also reduced. These results further establish that HsA inhibits the activity of PDK1 by interfering with the interaction between PDK1 and the L2 lipoamide domain of PDH-E2.

### HsA increases the mitochondrial reactive oxygen species (ROS) levels and induces apoptosis of DLD-1 cells

**Figure 4:**
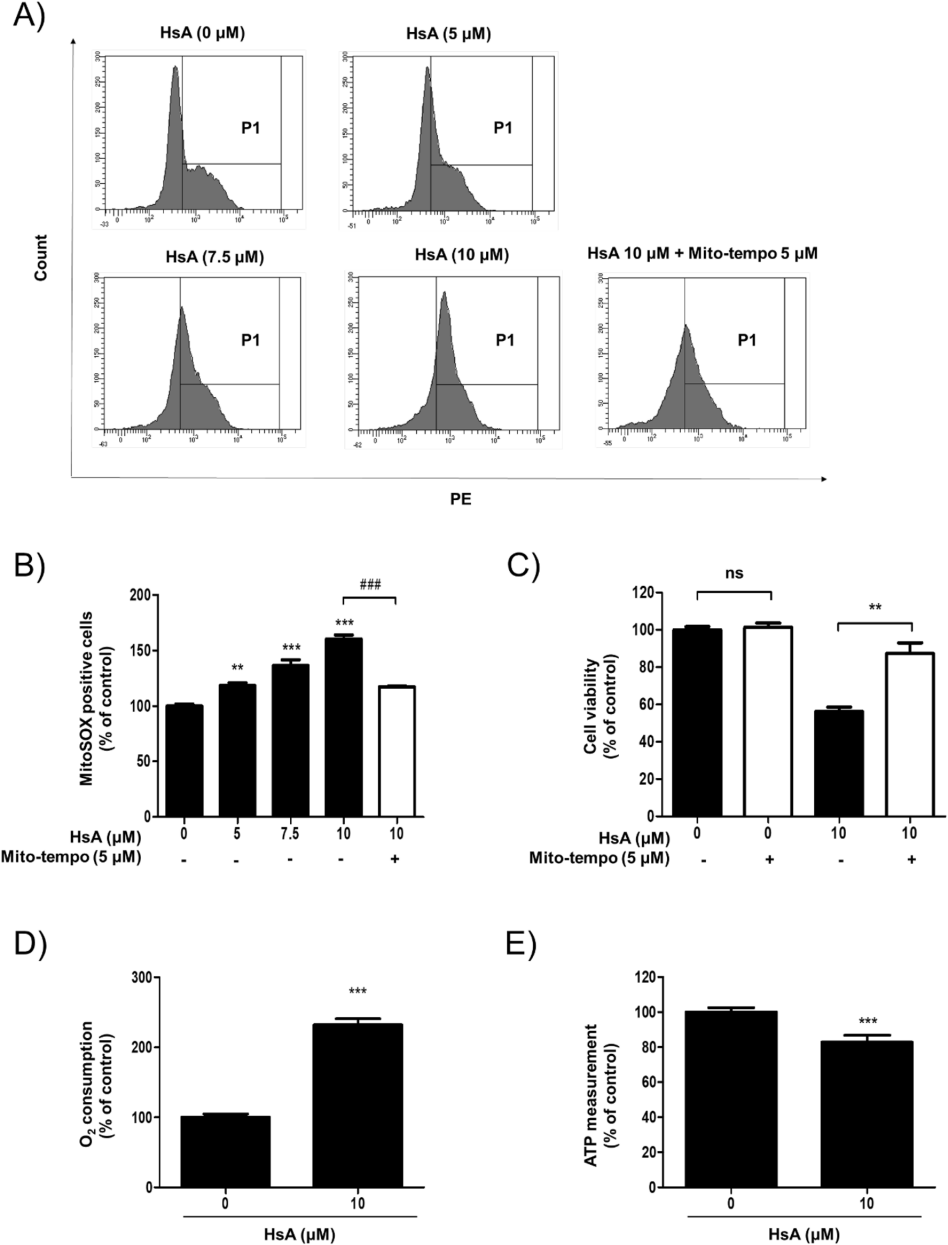
HsA increases the mitochondrial ROS level and oxygen consumption. (**A**) DLD-1 cells were treated with HsA (0, 5, 7.5, 10 μM) in the presence or absence of Mito-tempo (5 μM) for 12 h. Mitochondrial superoxide was detected by MitoSOX staining and analyzed by FACS. (**B**) The intensity of MitoSOX positive cells were measured and indicated as a percentage of control. (**C**) DLD-1 cells were treated with HsA (0, 10 μM) in the presence or absence of Mito-tempo (5 μM) for 24 h. The cytotoxic effects of HsA were measured by MTT Assay. The results (**B**, **C**) are shown as mean + SEM. **p < 0.01; ***p < 0.001 compared with the control group (1st lane). ^###^p < 0.001 compared to the positive control (4th lane). (**D**) DLD-1 cells were treated with HsA (0, 10 μM) for 12 h. Oxygen consumption rate was determined using a commercially available kit. The percentage values were calculated as compared to the control, and are presented as mean + SEM. ***p < 0.001 compared with the control group. (**E**) DLD-1 cells were treated with HsA (0, 10 μM) for 12 h. ATP level was determined using a commercially available kit. The percentage values were calculated as compared to the control, and are presented as mean + SEM. ***p < 0.001 compared with the control group.

**Figure 5:**
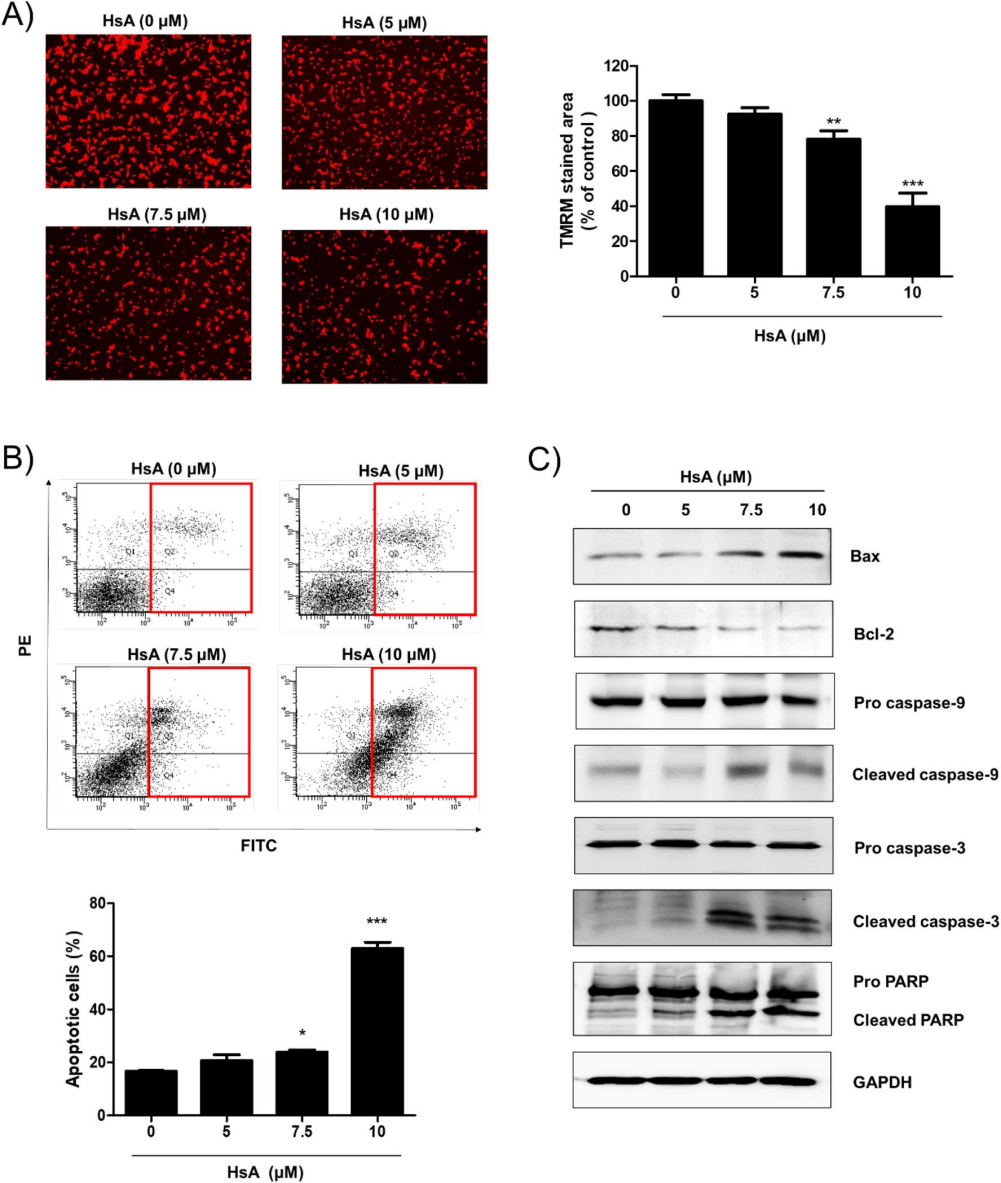
HsA induces apoptosis of DLD-1 cells. (**A**) The DLD-1 cells were treated with HsA (0, 5, 7.5, 10 μM) for 24 h. Intact mitochondria was detected by TMRM staining and analyzed by microscopy. The intensity of fluorescent was measured and indicated as a percentage of control. (**B**) DLD-1 cells were treated with HsA (0, 5, 7.5, 10 μM) for 24 h. The cells were stained with Annexin V and PI, and the number of apoptotic cells was measured by FACS. (**C**) DLD-1 cells were treated with indicated concentrations of HsA (0, 5, 7.5, 10 μM) for 24 h. The protein levels of Bax, Bcl-2, caspase-3, caspase-9, and PARP were examined by Western blot analysis. GAPDH expression was used as an internal loading control. The results (**A**, **B**) are shown as mean ± SEM. *p < 0.05; **p < 0.01; ***p < 0.001 compared with the control group.

PDK1 inhibition induces mitochondrial-pathway apoptosis via increased phosphorylation byproducts such as ROS^19,20^. Thus, to determine whether the cytotoxic effects of HsA are associated with the mitochondrial ROS production, DLD-1 cells were treated with Mito-tempo, a mitochondria-targeted antioxidant, and mitochondria ROS was measured by Mito-sox assay. As shown in Fig. 4A, mitochondria ROS levels were increased by HsA treatment. The mitochondrial ROS production and cytotoxicity induced by HsA treatment were rescued by Mito-tempo treatment (Fig. 4B,C). O_2_ consumption rate was also improved by HsA treatment (Fig. 4D). In the same condition, the increase of the O_2_ consumption rate and the reduction of phosphorylation of PDHA were higher than that treated with a well-known PDK inhibitor, DCA (Fig. S5). The intracellular ATP level was decreased by treatment with HsA (Fig. 4E). As a consequence of increased mitochondrial ROS, HsA also collapsed the mitochondrial membrane potential (Fig. 5A) and induced apoptotic cell death (Fig. 5B) in DLD-1 cells. The Bax/Bcl-2 ratio, an important marker of mitochondrial membrane depolarization, was also increased by HsA treatment (Fig. 5C). Finally, HsA increased the activation of caspase-9, caspase-3, and PARP, indicating the activation of the mitochondrial pathway for apoptosis. To validate the specificity of HsA for PDK1, pLKO.1, and PDK1 silencing DLD-1 cells were used (Fig. S5D)^21^. The anti-tumor effect of HsA was suppressed in PDK1 silencing DLD-1 cells (Fig. S5E). All these results indicate that HsA induces apoptosis of colon cancer cells through ROS-mediated mitochondrial damage.

### HsA reduces in vivo tumor growth in the allograft model

**Figure 6:**
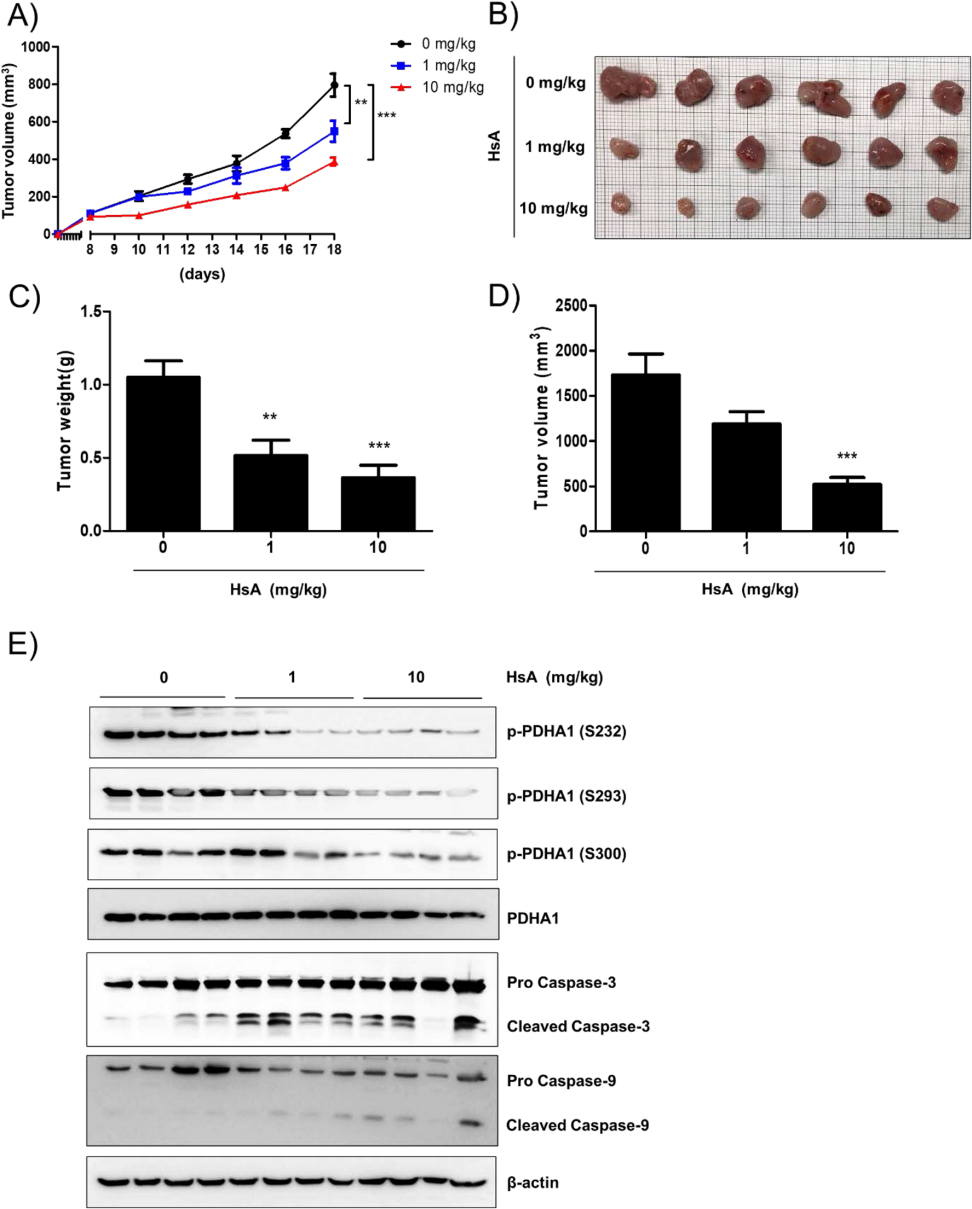
Anti-tumor activity of HsA in BALB/c mice bearing CT26 cells. CT26 cells were separated and suspended in PBS (5 × 10^6^ cells/100 μL of PBS) and subcutaneously injected into the dorsal of 6-week-old BALB/c mice. The control group mice were injected 100 μL of corn oil and the HsA-treated groups were injected HsA (1 mg/kg, 10 mg/kg in 100 μL of corn oil/mice) once a dayfrom 8th day using abdominal injection for10 days. (**A**) The tumor volume of each mouse was measured every other day during the injection of HsA. (**B**) The tumor tissues were excised, and the images were taken. The weight (**C**) and volume (**D**) of the tumors in each group were measured. The results (**C**, **D**) are shown as mean ± SEM. *p < 0.05 and ***p < 0.001 compared with the control group. (**E**) The tumor tissues were lysed and the amounts of phosphorylated PDHA1(S232, S293,and S300), total PDHA1, caspase-3, and caspase-9 were measured by Western blot analysis. β-actin expression was used as a loading control.

To further confirm the effect of HsA we have seen in vitro, we injected a murine colorectal cancer cell line, CT26 cells, into immunocompetent BALB/c mice to create an allograft CRC model. From the 8th day of tumor injection, HsA (0, 1, 10 mg/kg/day) was treated for 10 days. The average tumor size when HsA started to treat was about 111.85 mm^3^. Tumor volume was suppressed by HsA (Fig. 6A), but HsA did not cause significant weight loss (Fig. S6A). Then, the tumor tissue specimens were removed from the sacrificed mice, and images were taken (Fig. 6B). HsA showed a significant effect in suppressing the growth of CT26 cells in a dose-dependent manner as seen by the reduced tumor volume and weight (Fig. 6D–E). Finally, we measured the activity of HsA on PDK1 in animal models, the phosphorylation of PDHA1 was determined by Western blot analysis. Consistent with the in vitro experiments, HsA decreased the phosphorylation of PDHA1 in allograft tumor tissues in a dose-dependent manner. Moreover, activation of caspase-9 and caspase-3 was also induced by treatment with HsA (Fig. 6F). Considering there little or less hepatocellular or renal toxicity, HsA suppressed the growth of CT26 cells by reducing PDK1 activity (Fig. S6B–D). These results suggest that HsA has an inhibitory effect on PDK1 activity and consequently reduces the growth of CT26 cells in the murine allograft model.

## Discussion

In this study, we focus on targeting PDK1 and suppressing the growth of glycolytic tumors. We showed that HsA reduced the growth of several CRC cells by suppressing the kinase activity of PDK1 at both in vitro and in vivo levels. HsA interfered with the binding between PDK1 and the L2 lipoamide domain of PDH-E2, consequently dephosphorylated and activated the PDHA1. The activated PDH complex upon HsA treatment rewired the cancer metabolism from lactic fermentation to OXPHOS, thereby increased the ROS production and mitochondrial depolarization, and ultimately resulted in apoptotic cell death (Fig. 6E). These data demonstrated a good correlation with previous studies that PDK1 is regarded as a crucial point in tilting the energy balance in the favor of cancer cells^22,23^. Furthermore, PDK1 inhibitors were used in co-treatment with other drugs, as previously shown that inhibition of PDK1 improves the anti-cancer effects of EGFR tyrosine kinase inhibitors^24^.

Inhibition of PDKs increases the mitochondrial production of the electron-donor NADH, a substrate of the ETC complex I, causing complex I dysfunction in cancer cells^23^. Once the O_2_ consumption is uncoupled with ATP synthesis, the oxidative stress increases significantly and produces cellular damages^25,26^. The knockdown of PDK1 could also increase mitochondrial oxygen consumption and inhibit tumor proliferation by activating apoptosis^11,27^. However, PDK1 is reported to be expressed at low levels in most of the normal tissues and energy consumption in normal cells relies mainly on mitochondrial OXPHOS^4,28^. Thus, inhibition of PDK1 would not cause serious problems in normal cells.

Crystal structure studies revealed that the three domains of PDKs, including the pyruvate-binding domain (N-terminal regulatory domain), lipoamide-binding domain, and nucleotide-binding domain (C-terminal catalytic domain), are important for the regulation of PDK activity^29,30^. PDKs are engaged by the PDH complex by precise binding to the inner lipoamide domain of the PDH-E2/E3 binding protein (E3BP) core, and they effectively phosphorylate the PDHA1^31^. PDK1 strongly binds to the L2 lipoamide domain of PDH-E2 and more weakly to the L1 lipoamide domain. The L3 domain of E3BP does not interact with PDK1^29^. In this study, the computational prediction and biochemical assay demonstrated that HsA interfered with the binding of PDK1 to the L2 lipoamide domain of PDH-E2. This mode of action is similar to that of the other established PDK inhibitors, including AZD7545 and Nov3r^18,29^. However, HsA does not share the characteristics in chemical structure with these established inhibitors of the lipoamide-binding domain.

Several established small molecule inhibitors of PDK1 have been developed. For example, AZD7545^32^, Nov3r^33^ targets the lipoamide-binding pocket; radicicol^34^, VER-246608^35^, and JX06^36^ targets the ATP-binding pocket; DCA^37^ binds to pyruvate-binding pocket. However, very limited PDK1 inhibitors are now developing as an anticancer drug. No studies have investigated the anticancer effect of AZD7545, Nov3r, or VER-246608. DCA and JX06 have been reported to reduce cancer growth through modulating metabolism and inducing apoptosis^36,38^. Recently, compound 10, a novel PDK inhibitor targeting lipoamide-binding pocket has successfully reduced the growth of several cancer cells^39^. In the present study, we could not precisely determine the in vitro inhibitory efficacy of HsA on the PDK1 activity. However, the IC_50_ of HsA on cancer growth is comparable to that of previously established PDK inhibitors, such as DCA (over 10 mM), JX06 (4 μM to HT-29 cells), and compound 10 (45.35 μM to HCT-116 cells)^36,38,39^. Also, typical oral and parenteral daily doses of DCA or JX06 was ranged from 10 to 50 mg/kg or 40 to 80 mg/kg, respectively^36,40^. In vivo efficacy of compound 10 was not tested. In this study, HsA could suppress significantly the growth of CT26 cells at the concentration of 10 mg/kg.

However, this study has several limitations as follows. First, this study adopted an allograft cancer model using only one CRC cells. To ensure the in vivo anti-cancer efficacy of HsA, a further extensive study using various types of human cancer cells using in vivo xenograft model should be performed. Second, like other natural products^41^, HsA might bind to the other molecular targets, such as previously reported Nrf2 and NF-κB in inflammation and hepatic fibrosis^42,43^, and AMPK in liver cancer^44^. AMPK could be regulated by Acetyl-CoA or ATP production which is regulated by PDK inhibitors^45,46^. AMPK also might regulate the expression or activity of PDKs^47,48^. To elucidate the relationship between AMPK and PDK regulation by HsA, further extensive molecular experiments should be conducted. Furthermore, to verify the possibility of developing an anticancer drug, the in vivo safety and pharmacological property of HsA also should be examined in further studies. Despite these limitations, we suggest that HsA might be a potent candidate for developing anti-metabolic cancer drugs because HsA has a comparable in vivo efficacy and a unique structural feature compared to previously established PDK1 inhibitors. Natural products could be a valuable starting point for drug discovery by enhancing the efficacy and target specificity^49^.

In summary, HsA suppresses the growth of CRC cells in both in vitro and in vivo models by inhibiting the PDK1 activity, thereby enhancing the metabolic shift from glycolysis to OXPHOS, and consequently inducing mitochondrial ROS-mediated apoptosis. We also confirmed that HsA achieves its inhibitory action on PDK1 by targeting its lipoamide-binding pocket. Although more pharmacological studies and clinical evidence are required, HsA has shown a potential candidate as a novel PDK1 inhibitor for colorectal cancer treatment.

## Methods

### Antibodies and reagents

Antibodies used for western blotting are indicated in Table S1. NAC was purchased from Amresco (Solon, OH, USA). 3-(4,5-Dimethylthiazol-2-yl)2,5-diphenyltetrazolium bromide (MTT), oligomycin, and DCA were supplied by Sigma-Aldrich (St. Louis, MO). All the other chemicals were provided by Sigma-Aldrich unless otherwise indicated. HsA used in this study was a deposit from our previous study^16^.

### Cell culture

Human colon cancer cell lines, SW480 and RKO, were obtained from American Type Culture Collection (Manassas, VA). The other cells were supplied by Korean Cell Line Bank (Seoul, Korea). All the complete growth media were supplemented with 10% heat-inactivated fetal bovine serum (FBS; Sigma-Aldrich) and 1% penicillin/streptomycin (Gibco, Rockville, MD). The cells were incubated in a cell culture incubator at 37 °C in 5% CO_2_ and 100% humidity conditions.

### Cell viability assay

Cell viability was examined using the MTT assay. The cells were seeded on 24-well culture plates (1 × 10^5^ cells/well) and then treated with various concentrations of drugs for 24 h or 48 h. MTT solution (2.0 mg/mL) was added to each well of the plates and the culture plates were incubated for 4 h at 37 °C and 5% CO_2_ conditions. The culture medium was removed and the number of formazan crystals formed in the living cells was determined by measuring the absorbance at 540 nm with a Spectramax M2 microplate reader (Molecular Devices, San Jose, CA, USA).

### Lactate production assay

Lactate production in DLD-1 cells was measured with a lactate fluorometric assay kit (Biovision, CA, USA), as previously described^50^.

### LDHA activity assay

LDHA activity was determined by measuring the NADH oxidation as described in a previous study^51^. Recombinant LDHA protein (10 ng) was used for the in vitro LDHA assay and 1 μg of total protein from the cell lysates was used for the intracellular LDHA assay. Various concentrations of HsA were added into the reaction solution containing 20 μM NADH, 20 mM HEPES (pH 7.2), 2 mM pyruvate, and 0.05% bovine serum albumin (BSA). In the case of the intracellular assay, HsA was added to the cells. The amount of NADH was measured using the Spectramax M2 spectrofluorometric (Molecular Devices) with 340 nm/360 nm of excitation/emission wavelengths. The activity of LDHA was inversely calculated from the amount of NADH consumption.

### PDK1 substrate binding assay

GST and GST-PDK1 construct (obtained from Professor Jing Chen, Emory University) were transfected into HEK 293 T cells using polyethylenimine. After 24 h of transfection, cells were treated with 10 μM of HsA. Then, the cells were harvested and lysed. GST and GST-PDK1 were pulled down using Glutathione Sepharose 4B beads (Amersham Bioscience). The beads were washed with 20 mM potassium phosphate (pH 7). The samples were run on an SDS-PAGE gel, followed by immunoblotting.

### Oxygen consumption assay

Oxygen consumption in DLD-1 cells was measured with an oxygen consumption rate assay kit (Cayman Chemical, Ann Arbor, MI). DLD-1 cells were seeded in 96-well tissue culture plates at a density of 4 × 10^4^ cells and the experiment was conducted according to the method provided.

### ATP bioluminescence assay

ATP activity in DLD-1 cells was measured with an ATP Bioluminescence Assay Kit (Sigma-Aldrich). DLD-1 cells were seeded in 96-well tissue culture plates at a density of 4 × 10^4^ cells and treated with or without HsA for 12 h. The experiment was conducted according to the method provided.

### Measurement of mitochondrial ROS

The production of mitochondrial ROS in cells was determined using MitoSOX Red (Invitrogen, Karlsruhe, Germany). In brief, 1 μM of MitoSOX Red was added to the cells cultured in a conditioned medium and incubated at 37 °C for 10 min. The fluorescence intensity was analyzed using a BD FACS Canto II by measuring the excitation/emission at 510/580 nm wavelengths.

### Mitochondrial depolarization assay

Pre-treated DLD-1 cells were incubated with 250 nM TMRM (Thermo Fisher Scientific) for 30 min. The cells were washed with phosphate-buffered saline (PBS). The fluorescent images of the samples were observed and captured using a fluorescence microscope (Zeiss AX10 Imager.M1; Carl Zeiss Microimaging, Oberkochen, Germany) with excitation/emission at 535/600 nm wavelengths. To quantify the mitochondrial membrane potential, the fluorescence intensity of each captured image was calculated using Image J (NIH, MD).

### Detection of apoptotic cells

Apoptotic cells were examined using an annexin V-FITC apoptosis detection kit (Life Technologies, Carlsbad, CA). The cells (5 × 10^5^) were resuspended in 500 µL of binding buffer and incubated with 5 µL of annexin V-FITC and propidium iodine (PI) for 15 min at room temperature. The fluorescence intensities of the samples were examined using a BD FACS Canto II flow cytometer by measuring the annexin V-FITC excitation/emission at the wavelengths of 494/518 nm and PI at the wavelengths of 535/617 nm.

### Animals and tumor allografts

Male BALB/c mice (6-weeks-old, weight 20–24 g, n = 8 per group) were obtained from Orient Bio Inc. (Sungnam, Korea). All the experimental procedures followed the Guidelines for the Care and Use of Laboratory Animals of the National Institutes of Health of Korea and were approved by the Institutional Animal Care and Use Committee of Pusan National University, Pusan, Republic of Korea (protocol number: PNU-2017-1603). Tumor tissue specimens were immediately removed from the mice and the volumes of tumor tissues were measured with a pair of calipers and were calculated according to the formula [(length × width^2^)/2]. Murine serum was collected to measure the hepatocellular and renal cytotoxicities using commercial biochemistry analyses from Green Cross Co. (Seoul, Korea).

### Statistical analysis

The values from the cell viability, LDH assay, lactate production, and in vivo studies were calculated as percentage values in comparison to the control group and expressed as mean ± standard error of the mean (SEM). The results from the experiments estimating oxygen consumption and ATP rate, mitochondrial membrane potential, and apoptotic cells were calculated as fold change in comparison to the control. Hepatocellular and renal toxicity of the mice were analyzed by one-way analysis of variance with a post hoc Tukey's comparison using GraphPad Prism software (GraphPad Software, San Diego, CA). The minimum level of statistical significance was set at a p-value of 0.05 for all the analyses. All the experiments were independently conducted three times, except for the animal studies. Further details were shown in the supplementary material.

## Supplementary Information

Supplementary Information.
