# Supplementary material for: Hemistepsin A suppresses colorectal cancer growth through inhibiting pyruvate dehydrogenase kinase activity
Source: Sci Rep. 2020 Dec 14;10:21940. doi: 10.1038/s41598-020-79019-1 (PMC7736850; doi:10.1038/s41598-020-79019-1)
Supplement: Supplementary file 1 — Supplementary Information. [file 41598_2020_79019_MOESM1_ESM.pdf]

Supplementary Information

Hemistepsin A Suppresses Colorectal Cancer Growth through Inhibiting Pyruvate Dehydrogenase Kinase Activity

Ling Jin[a,b], Eun-Yeong Kim[a,b], Tae-Wook Chung[b], Chang Woo Han[c], So Young Park[c], Jung Ho Han[b], Sung-Jin Bae[b], Jong Rok Lee[d], Young Woo Kim[e], Se Bok Jang[c], Ki-Tae Ha[a,b]*

[a]Department of Korean Medical Science, School of Korean Medicine,

[b]Healthy Aging Korean Medical Research Center, Pusan National University, Yangsan, Gyeongsangnam-do 50612, Republic of Korea

[c]Department of Molecular Biology, College of Natural Sciences, Pusan National University, Geumjeong-gu, Busan 46241, Republic of Korea

[d]Department of Pharmaceutical Engineering, Daegu Haany University, Gyeongsan, Gyeongsangbuk-do 38610, Republic of Korea

[e]School of Korean Medicine, Dongguk University, Gyeongju, 38066, Korea

*Corresponding Author

Ki-Tae Ha, Department of Korean Medical Science, School of Korean Medicine, Pusan National University. Busandaehak-ro 49, Yangsan, Gyeonsangnam-do, 50612, Republic of Korea.

Tel: +82-51-510-8464; Fax: +82-51-520-8420

E-mail: hagis@pusan.ac.kr

Supplementary material

Western blot analysis. Total protein was extracted from the cells or tissues using 1% NP-40 lysis buffer containing 150 mM NaCl, 10 mM HEPES (pH 7.45), 1% NP-40, 5 mM Na-pyrophosphate, 5 mM NaF, 2 mM Na_3VO_4, and protease inhibitor cocktail (Roche Applied Science, Mannheim, Germany). The same amount of protein from all the samples was separated by sodium dodecyl sulfate-polyacrylamide gel electrophoresis (SDS-PAGE). Then, the proteins were transferred from the gel onto a 0.45 µm NC nitrocellulose blotting membrane (Amersham Bioscience, Uppsala, Sweden) by electroblotting. The bands of the target proteins were detected using ECL Plus (Amersham Bioscience) and Image Quant LAS 4000 (GE Healthcare, Chicago, USA). The whole blot used in this study was shown in Figure S8.

Structural prediction of the PDK1 and HsA interaction. The crystal structure of PDK1 protein was downloaded from the Protein Data Bank (ID: 2Q8F) (http://www.rcsb.org/pdb/home/home.do) server and the 2D structure of HsA (CID: 10043230) compound was obtained from the NCBI PubChem Compound database (Figure 1A). The structure of HsA was converted to an energy-minimized structure by using OpenBabel in Pyrx and Research Design was used to computationally determine the potential activity and the binding affinity of HsA to PDK1 protein. The structural prediction of the PDK1 and HsA complex was performed using AutoDock Vina in Pyrx. Among the complex models, the structure having the smallest binding energy was chosen. The binding affinity of the HsA to PDK1 was -6.9 kcal/mol and the docking result was visualized using PyMOL.

Isothermal titration calorimetry. ITC measurements were carried out using MicroCal Auto_iTC200 (GE healthcare) at 25°C. The purified PDK1 protein was dialyzed in the buffer

containing 50 mM Tris-HCl (pH 8.0) and 200 mM NaCl at a concentration of 0.1 mM and HsA was solubilized in the same buffer at a concentration of 1 mM. One percent DMSO was added to the ITC buffer for the titration of HsA. The raw data were integrated and normalized using MicroCal Origin 7.0 software (OriginLab Co., Northampton, MA), resulting in a plot of ΔH (mol of injectant)$^{-1}$ versus molar ratio. The determined K_A and ΔH values were used to calculate ΔS from the standard thermodynamic equation. For lipoamide-binding analysis, the PDK1 protein was dialyzed in buffer A (50 mM Tris-HCl at pH 7.5 and 200 mM NaCl) at a concentration of 0.1 mM. The ligands (L1, L2, and HsA) were solubilized in the same buffer at a concentration of 1.5 mM. Titrations were conducted with 20 injections and injected at 150 sec intervals and the stirring speed was 1,000 rpm. The determined K_a and ΔH values were used to calculate ΔS from the standard thermodynamic equation. ITC experiments were performed with MicroCalorimeter AutoITC200 (GE healthcare) at 25 ℃ and the data were analyzed using MicroCal Origin software. L1 and L2 domains (Supplementary Table 3) were synthesized at Bioneer (Seoul, Korea).

Colony formation assay. DLD-1 cells were seeded on 6-well culture dishes at a density of 5 × 10^3 and cultured for 14 days. Colony staining was performed by Ethidium Bromide solutions. The colonies were imaged and analyzed using the Gel Doc system (Bio-Rad, California), as previously described [1].

Lentiviral-mediated short hairpin RNA silencing PDK1. The shPDK1 clone was designed as described in a previous study [2]. shPDK1, psPAX2, and pMD2.G were transfected into HEK 293T cells using polyethylenimine to generate lentivirus supernatant. pLKO.1 vector was used as a positive control**.** After 48 h transfection, the supernatant was collected and filtered.

Beginning the day of infection, DLD-1 cells were treated with polybrene (8 μg/mL, Sigma-Aldrich) for 2 days. Then, cells were selected by puromycin (1 μg/mL, Sigma-Aldrich) for 48 hours.

Supplementary reference

1. Guda, K., L. Natale, and S.D. Markowitz, *An improved method for staining cell colonies in clonogenic assays.* Cytotechnology, 2007. **54**(2): p. 85-88.
2. Fan, J., et al., *Tyrosine phosphorylation of lactate dehydrogenase a is important for NADH/NAD+ redox homeostasis in cancer cells.* Molecular and cellular biology, 2011. **31**(24): p. 4938-4950.

Table S1. Information of antibodies used in this study.

Antibody name	Company	Catalog number	Dilution for Western blot analysis
Phosphor –PDHA1 (S232)	MERCK	AP1063	1:1000 in 5% skim milk
Phosphor –PDHA1 (S293)	Abcam	ab177461	1:1000 in 1% skim milk
Phosphor –PDHA1 (S300)	MERCK	AP1064	1:1000 in 1% skim milk
PDHA1	Santa Cruz	sc-377092	1:1000 in 1% skim milk
PDK1	Enzo	ADI-KAP-PK112	1:1000 in 5% skim milk
PDK2	Signalway antibody	#41330	1:1000 in 1% skim milk
PDK3	Novusbio	NBP1-32581	1:1000 in 5% BSA
PDK4	Signalway antibody	#38562	1:1000 in 5% skim milk
LDHA	Abcam	Ab84716	1:1000 in 5% skim milk
HIF1α	Cell Signaling	#14179S	1:1000 in 1% skim milk
GAPDH	Santa Cruz	sc-32233	1:1000 in 5% skim milk
GST	Santa Cruz	sc-138	1:1000 in 5% skim milk
PDH-E2	Santa Cruz	sc-271352	1:1000 in 5% skim milk
Bax	Novusbio	NB100-56095	1:1000 in 1% skim milk
Bcl-2	Novusbio	NB100-56098	1:1000 in 1% skim milk
Caspase-3	Cell Signaling	#9665s	1:1000 in 1% skim milk
Caspase-9	Cell Signaling	#9508s	1:1000 in 1% skim milk
PARP	Cell Signaling	#9542s	1:1000 in 1% skim milk
β-actin	Sigma	#A2066	1:1000 in 5% skim milk
Anti-mouse IgG	Invitrogen	#RJ240410	1:4000 in skim milk
Anti-rabbit IgG	Invitrogen	#SA245916	1:4000 in skim milk

Table S2. The half-maximal inhibitory concentration (IC_{50}) of HsA on several CRC cells and normal fibroblast.

**Cell lines**	**IC_{50} (μM)**
SW480	18.12 ± 0.2843
HT29	28.66 ± 0.2232
RKO	15.03 ± 0.2029
DLD-1	10.31 ± 0.5536
CT26	9.27 ± 1.497
Detroit 551	52.72 ± 9.042

Table S3. Sequences of synthesized L1 and L2 domains.

domain	Sequence
L1	GCTTCCAGGAGCCTGTGCAGAAGGAGTAGGAGGCGAAGCCGTTGCTGCTGGG<br>GTTGGTGCAGGTGCAGCTTGGGGGGTAGGTGCTGCACTAGAATCCAGTGTATA<br>GTTTTTAAATGCTTCAATATCTTCAGGCTTGCCAACTGTGATACAAATAATTGC<br>TCCGATTGGCACATCACGCGTACCTTCTGCGACTAATATTTTTGCCATATAGCA<br>TTCTTCCAGGCTTTCAAACCCTACAGTTGCTTTATCAGTTTCCACTTCTGCAATT<br>AAGTCACCTTCATTAATCTTGTCACCCTCTTTTTTTTCCCAACGAGCTATGGTGC<br>CCGCCTGCATTGTAGGTGACAGGGAAGGCAAAGGAACTTTCTGATGCGGGGG<br>AAGACT
L2	GGGAGTAGGAGCTAAAGGCTGTGGGGTTGGAGGAACAGCGGCAACAGGCGGT<br>GGCGTAGGAGGTGGCGCTTGCGGTTTTAAATCTGTTACTTCTGTTGGACGGTAA<br>TCAGCAAAGGCGCTAATGTCTGCTTCCTTTTCCACAATGATACACAAAGGGGT<br>TCCTAAAGGGACATCGCGTGTGCCTTCAGGCACTAAGATTTTTGCTAAATAAC<br>CCTCTTCCTGGACCTCAAAACCAATCGTCGCTTTATCAGTTTCTATTTCTGCTAA<br>TAAGTCACCTTCACTTAACTTCTCCCCCACTTTTTTTTCCCAACGCTGAACCGTG<br>CCCATAGTCATGGTAGGAGATAAGGCAGGAAGAAGTACTTGCATATGAGGCG<br>GATATGA

Figure S1.

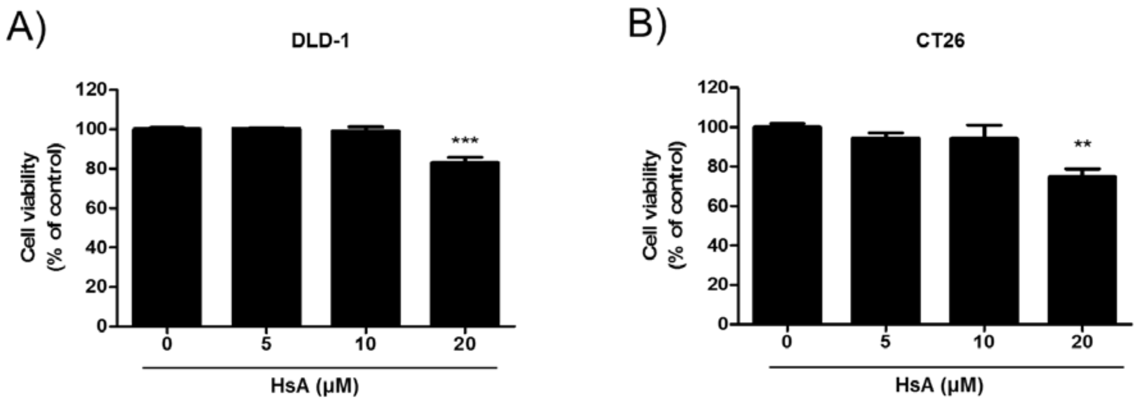

Figure S1. The cytotoxic effect of HsA. DLD-1 (A) and CT26 (B) cells are treated with indicated concentrations of HsA (0, 5, 10, 20 μM) for 4 h. The results were calculated to the percentage of control shown as mean ± SEM. **p<0.01 and ***p<0.001 compared with control group.

Figure S2.

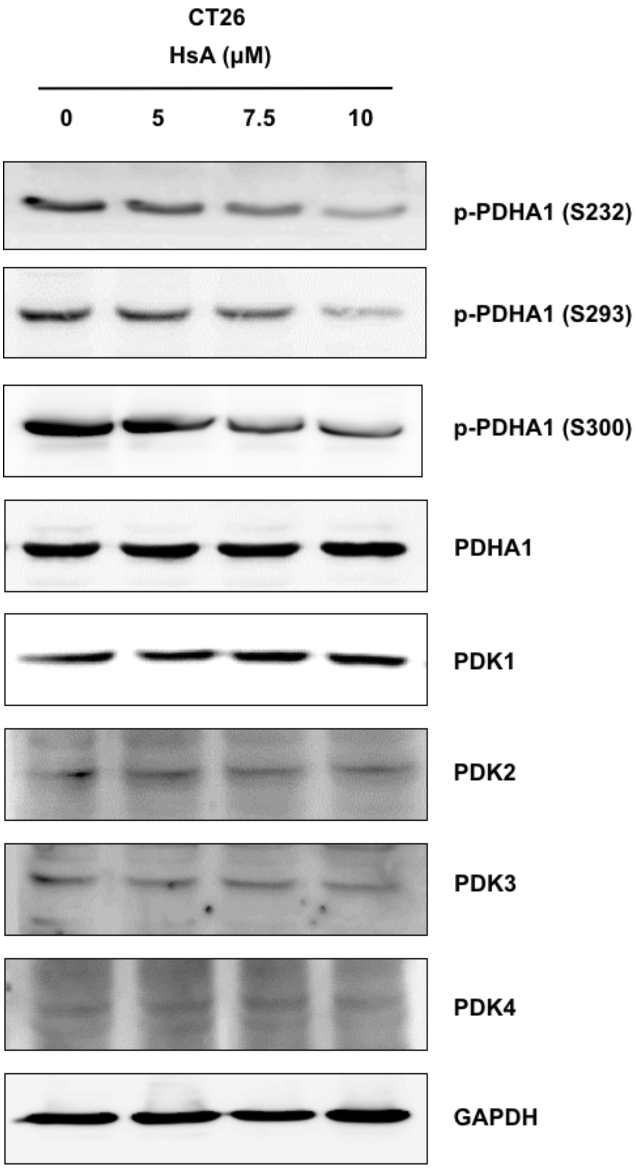

Figure S2. HsA inhibits the phosphorylation of PDHA1 in CT26 cells. CT26 cells were treated with indicated concentrations of HsA (0, 5, 7.5, 10 μM) for 4 h. The levels of phosphorylated PDHA1 (S232, S293, and S300), total PDHA1, and PDK1-4 were examined by Western blot analysis. GAPDH expression was used for internal control.

Figure S3.

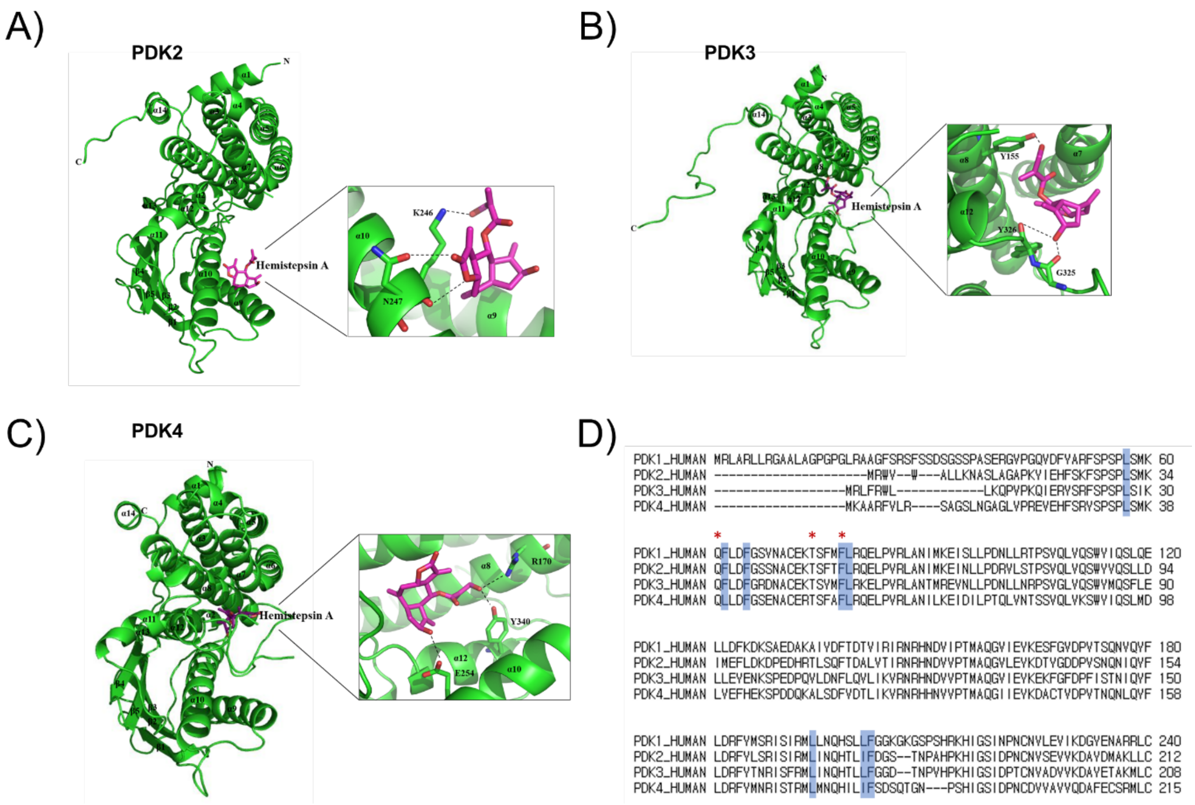

Figure S3. The interaction between HsA and PDKs (isoform 2-4). The modeled structure of PDK2 (A), PDK3 (B), and PDK4 (C) with HsA is shown as a ribbon representation. The interaction residues between PDKs and HsA are shown and hydrogen bonds are shown as black dotted lines. (D) Sequence alignments of human PDK isoforms from UniProt. Conserved residues participating in the lipoyl-binding pocket are marked with a blue box. The binding site of PDK1 and HsA is marked with *.

Figure S4.

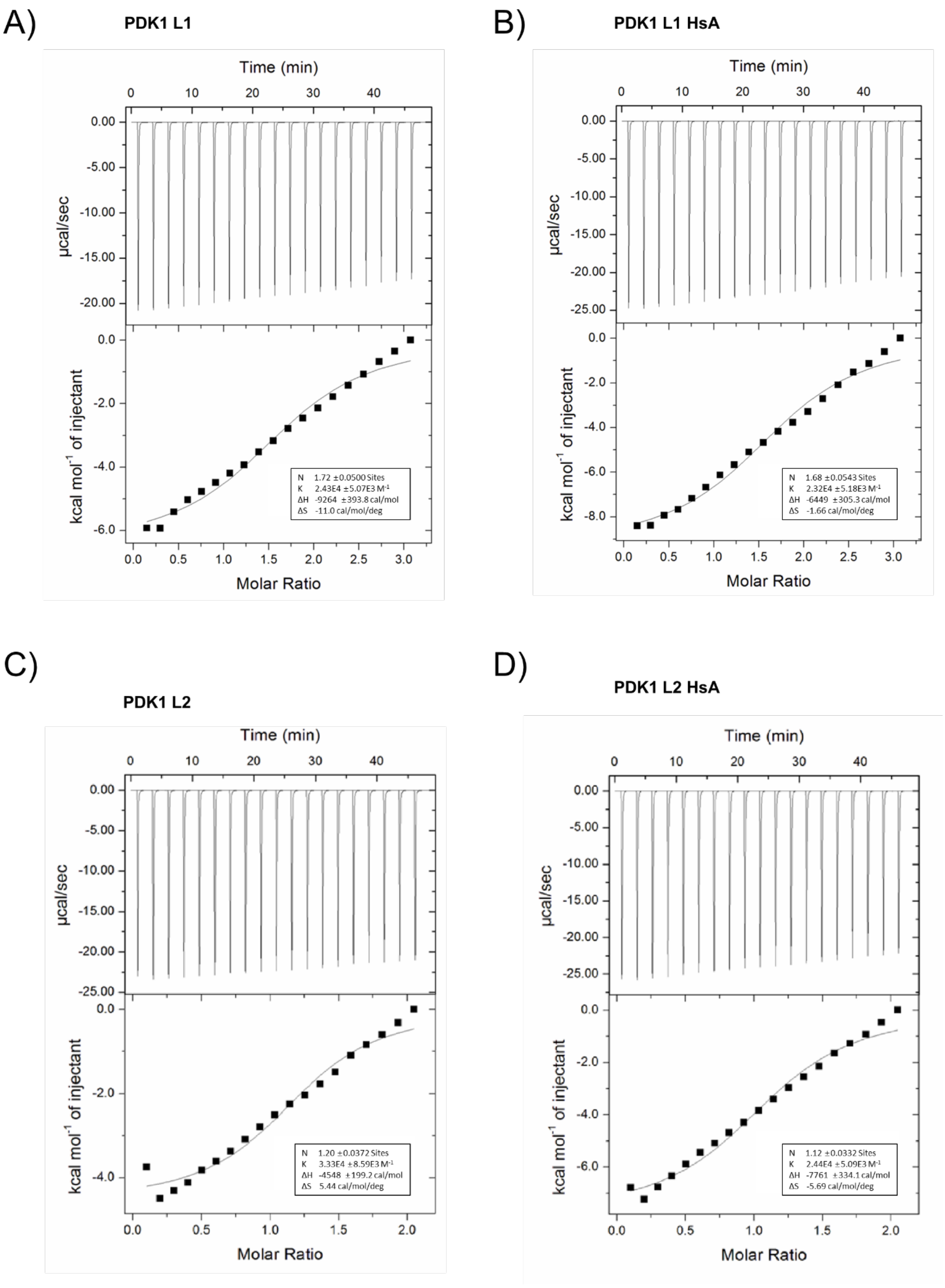
A)
PDK1 L1
Time (min)
µcal/sec
kcal mol-1 of injectant
N 1.72 ±0.0500 Sites
K 2.43E4 ±5.07E3 M-1
ΔH -9264 ±393.8 cal/mol
ΔS -11.0 cal/mol/deg
Molar Ratio
B)
PDK1 L1 HsA
Time (min)
µcal/sec
kcal mol-1 of injectant
N 1.68 ±0.0543 Sites
K 2.32E4 ±5.18E3 M-1
ΔH -6449 ±305.3 cal/mol
ΔS -1.66 cal/mol/deg
Molar Ratio
C)
PDK1 L2
Time (min)
µcal/sec
kcal mol-1 of injectant
N 1.20 ±0.0372 Sites
K 3.33E4 ±8.59E3 M-1
ΔH -4548 ±199.2 cal/mol
ΔS 5.44 cal/mol/deg
Molar Ratio
D)
PDK1 L2 HsA
Time (min)
µcal/sec
kcal mol-1 of injectant
N 1.12 ±0.0332 Sites
K 2.44E4 ±5.09E3 M-1
ΔH -7761 ±334.1 cal/mol
ΔS -5.69 cal/mol/deg
Molar Ratio

Figure S4. The binding affinities between PDK1 protein and ligands (L1, L1-HsA, L2, L2-HsA). (A-D) ITC analysis of the PDK1, and L1 (A), PDK1, L1, and HsA (B), PDK1 and L2 (C), PDK1, L2, and HsA (D) interaction is shown.

Figure S5.

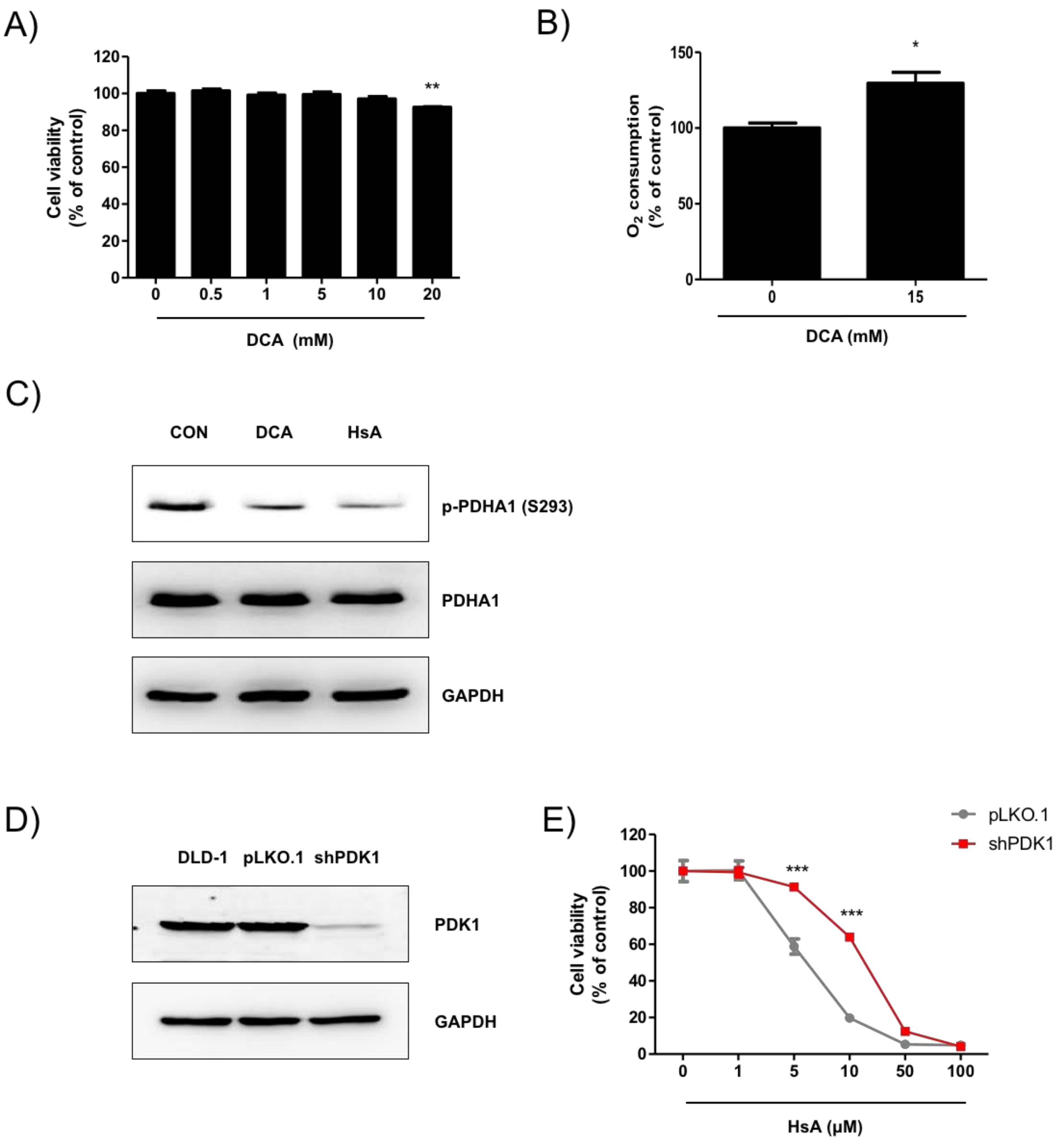

Figure S5. DCA, a well-known PDK inhibitor, increases oxygen consumption and acts as a positive control. (A) DLD-1 cells were treated with indicated concentrations of DCA (0, 0.5, 1, 5, 10, 20 mM) for 24 h. The cytotoxic effects of DCA were measured by MTT Assay. The result was shown as mean ± SEM. **p<0.01 compared with the control group. (B) DLD-1 cells were treated with DCA (15 mM) for 12 h. Oxygen consumption rate was determined using a

commercially available kit. The percentage values were calculated as compared to the control, and are presented as mean ± SEM. *, p<0. compared with the control group. (C) DLD-1 cells were incubated with DCA (15 mM) or HsA (10 μM) for 12 h. The levels of phosphorylated PDHA1 (S293) and total PDHA1 were examined by Western blot analysis. GAPDH expression was used as an internal loading control. (D) PDK1 silencing in DLD-1 cells by lentiviral short-hairpin RNA. pLKO.1 was used as a control. The expression of PDK1 was measured by Western blot analysis. GAPDH expression was used as an internal loading control. (E) PDK1 silencing DLD-1 cells and pLKO.1 control DLD-1 cells were treated with indicated concentrations of HsA (0, 1, 5, 10, 50, 100 μM) for 24 h. The cytotoxic effects of HsA were measured by MTT Assay. The results were calculated to the percentage of control shown as mean ± SEM. ***p<0.001 compared with control group.

Figure S6.

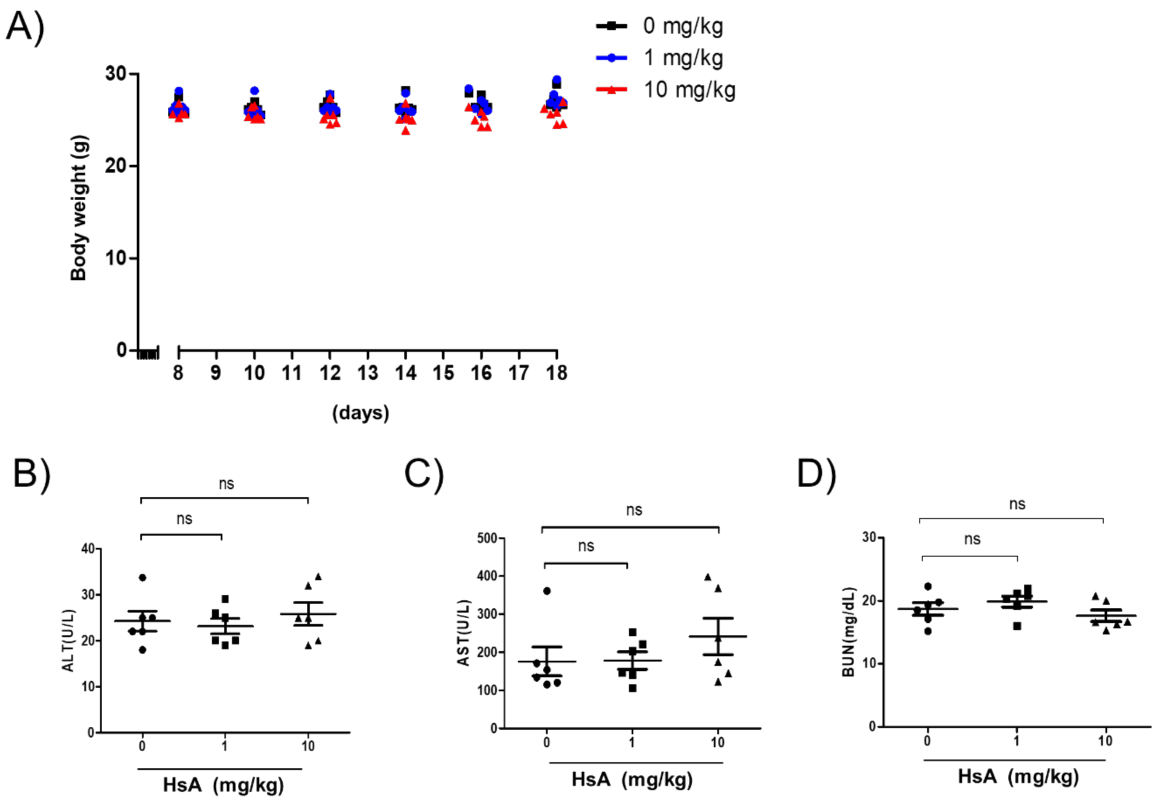

Figure S6. Hepatocellular and renal cytotoxicities of HsA. (A) The body weight of each mouse was measured every other day during injection of HsA. (B-D) Hepatocellular and renal cytotoxicities were analyzed by measuring murine serum ALT, AST, and BUN using commercial biochemistry. The results were compared with the control group by analysis of variance.

Figure S7.

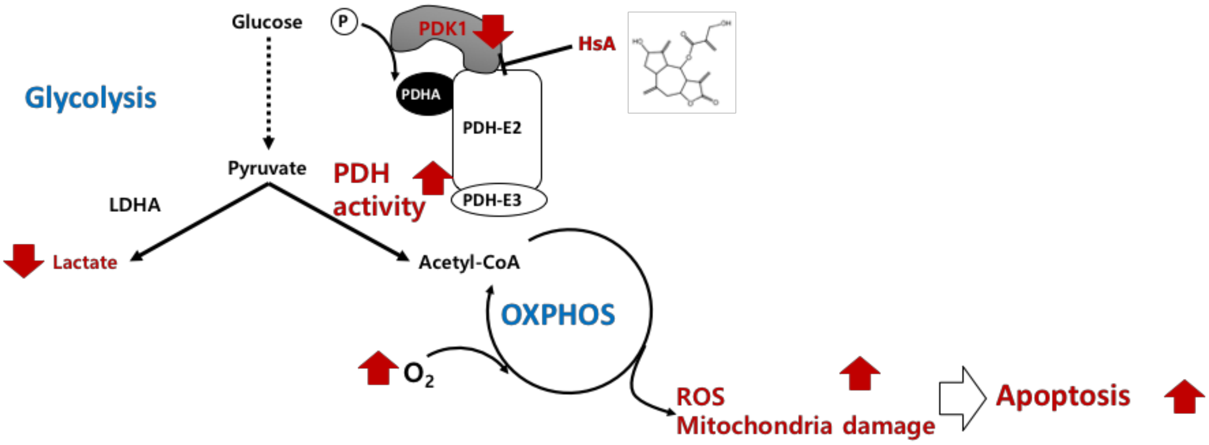

Figure S7. The role of HsA targeting colon cancer cells by inhibiting PDK1 activity. The figure shows that HsA reduced the growth of colorectal cancer through inhibition of PDK1 activity and consequent activation of mitochondrial ROS-dependent apoptotic pathway.

Figure S8.

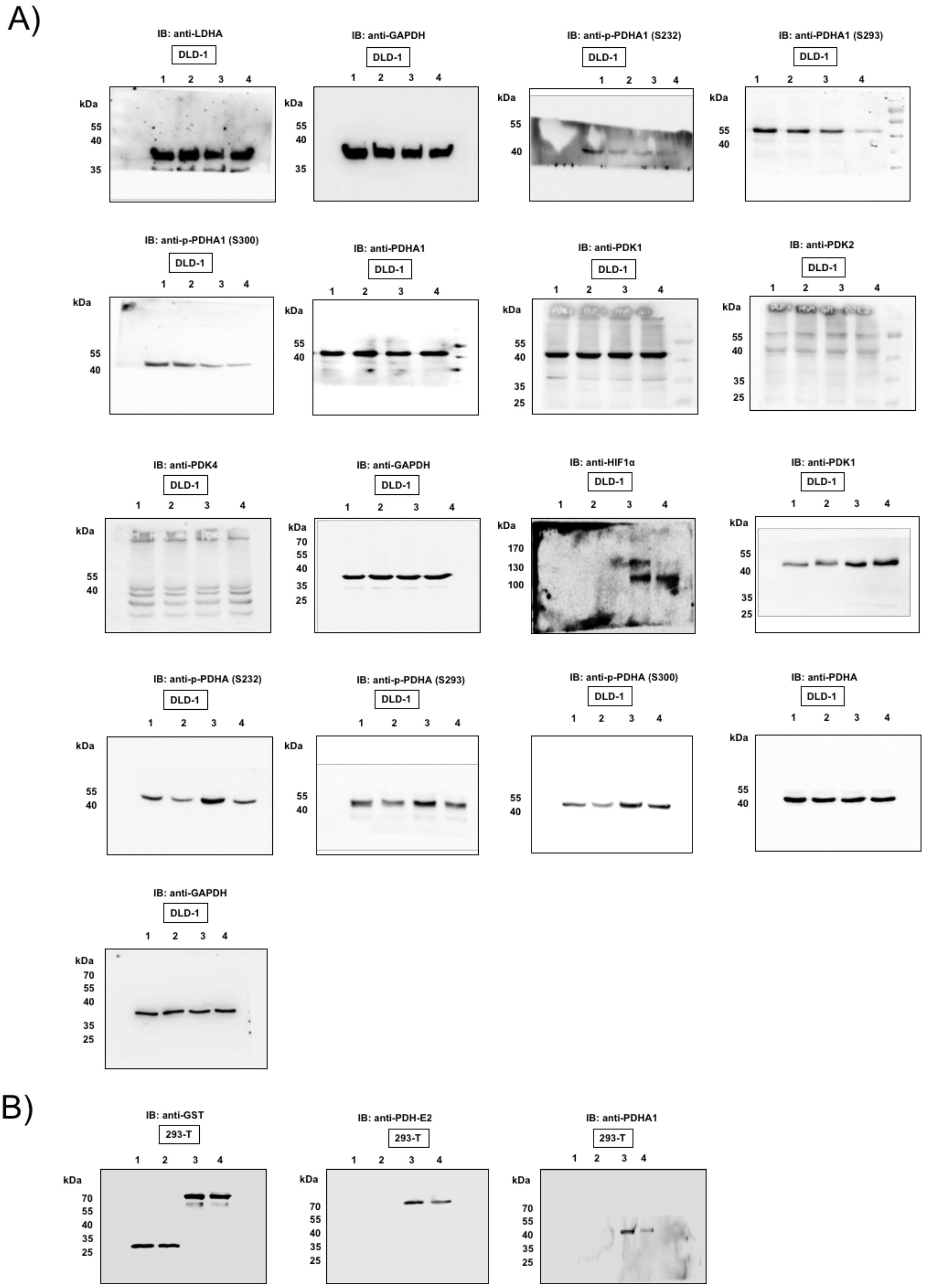
A)
IB: anti-LDHA
DLD-1
IB: anti-GAPDH
DLD-1
IB: anti-p-PDHA1 (S232)
DLD-1
IB: anti-PDHA1 (S293)
DLD-1
IB: anti-p-PDHA1 (S300)
DLD-1
IB: anti-PDHA1
DLD-1
IB: anti-PDK1
DLD-1
IB: anti-PDK2
DLD-1
IB: anti-PDK4
DLD-1
IB: anti-GAPDH
DLD-1
IB: anti-HIF1α
DLD-1
IB: anti-PDK1
DLD-1
IB: anti-p-PDHA (S232)
DLD-1
IB: anti-p-PDHA (S293)
DLD-1
IB: anti-p-PDHA (S300)
DLD-1
IB: anti-PDHA
DLD-1
IB: anti-GAPDH
DLD-1
B)
IB: anti-GST
293-T
IB: anti-PDH-E2
293-T
IB: anti-PDHA1
293-T
1 2 3 4
kDa
170
130
100
70
55
40
35
25

C)

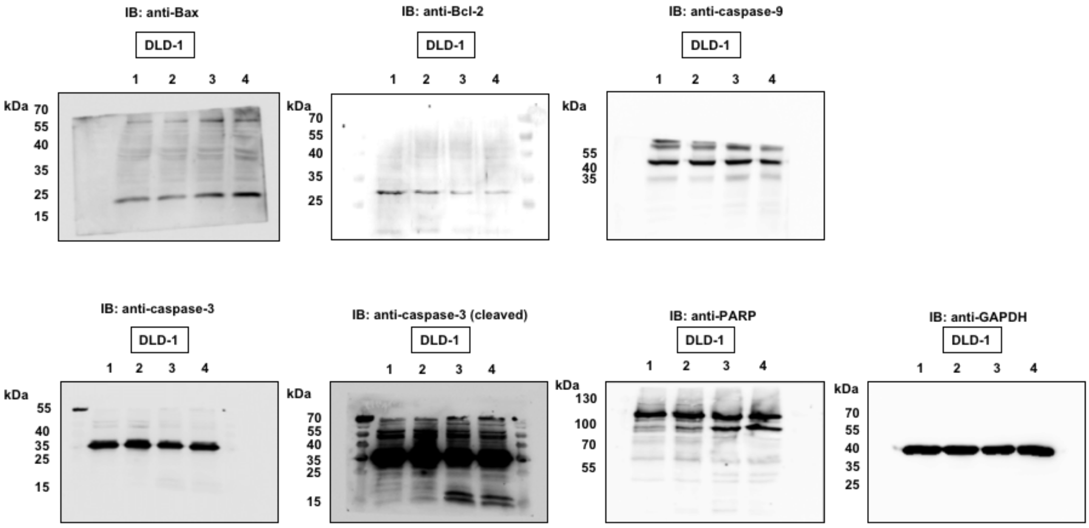
IB: anti-Bax
DLD-1
1 2 3 4
kDa 70 55 40 35 25 15
IB: anti-Bcl-2
DLD-1
1 2 3 4
kDa 70 55 40 35 25
IB: anti-caspase-9
DLD-1
1 2 3 4
kDa 55 40 35
IB: anti-caspase-3
DLD-1
1 2 3 4
kDa 55 40 35 25 15
IB: anti-caspase-3 (cleaved)
DLD-1
1 2 3 4
kDa 70 55 40 35 25 15
IB: anti-PARP
DLD-1
1 2 3 4
kDa 130 100 70 55
IB: anti-GAPDH
DLD-1
1 2 3 4
kDa 70 55 40 35 25

D)

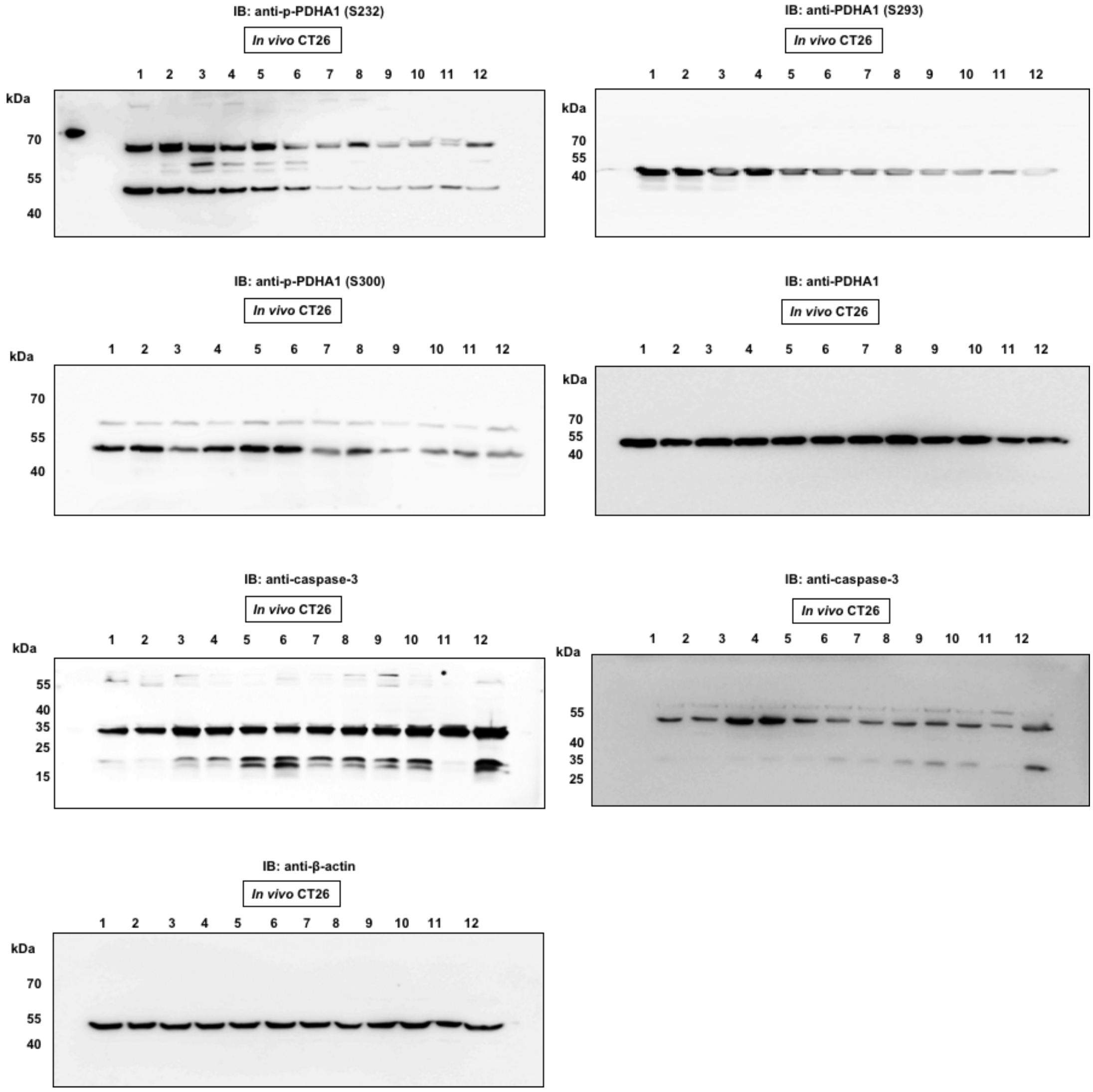
IB: anti-p-PDHA1 (S232)
In vivo CT26
1 2 3 4 5 6 7 8 9 10 11 12
kDa 70 55 40
IB: anti-PDHA1 (S293)
In vivo CT26
1 2 3 4 5 6 7 8 9 10 11 12
kDa 70 55 40
IB: anti-p-PDHA1 (S300)
In vivo CT26
1 2 3 4 5 6 7 8 9 10 11 12
kDa 70 55 40
IB: anti-PDHA1
In vivo CT26
1 2 3 4 5 6 7 8 9 10 11 12
kDa 70 55 40
IB: anti-caspase-3
In vivo CT26
1 2 3 4 5 6 7 8 9 10 11 12
kDa 55 40 35 25 15
IB: anti-caspase-3
In vivo CT26
1 2 3 4 5 6 7 8 9 10 11 12
kDa 55 40 35 25
IB: anti-β-actin
In vivo CT26
1 2 3 4 5 6 7 8 9 10 11 12
kDa 70 55 40

E)

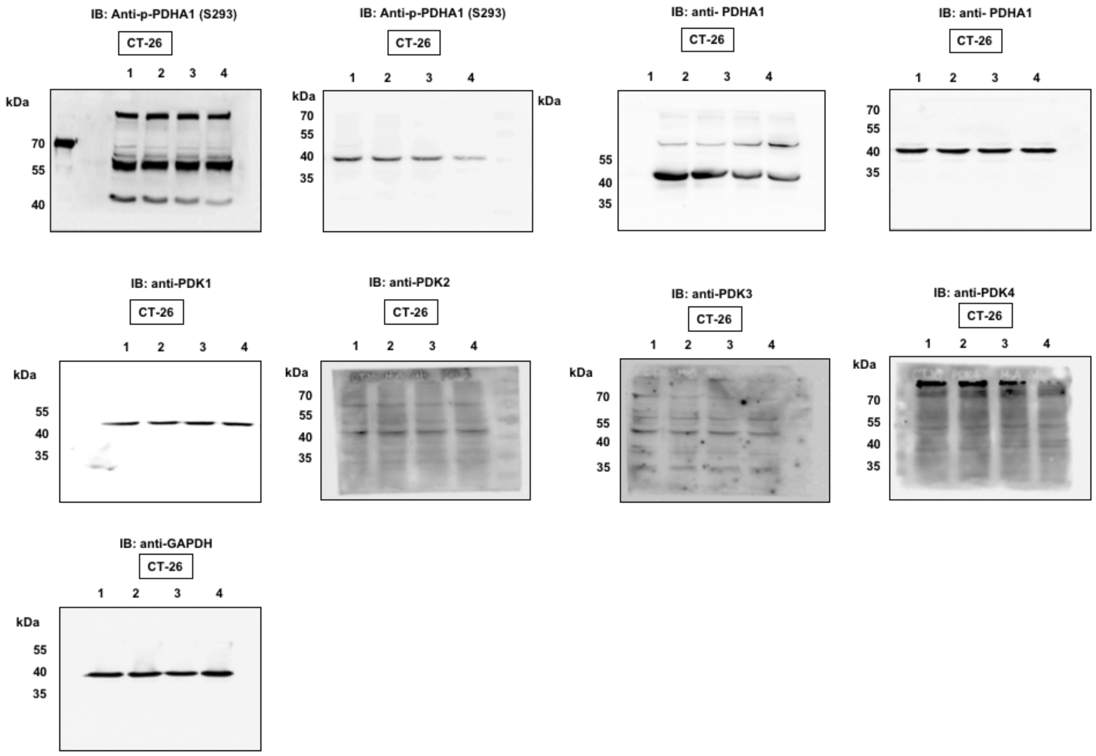

F)

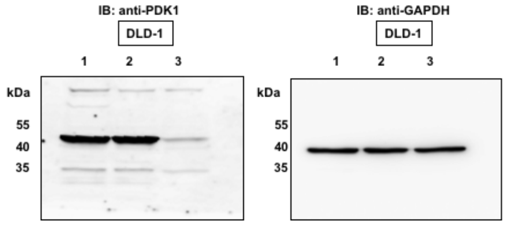

Figure S8. The whole blot used in this study. The figure shows the whole blot used in this study.
